# Unveiling the Potential of Rice Straw Nanofiber-Reinforced HDPE for Biomedical Applications: Investigating Mechanical and Tribological Characteristics

**DOI:** 10.3390/jfb14070366

**Published:** 2023-07-12

**Authors:** Mohamed Taha, Ahmed Fouly, Hany S. Abdo, Ibrahim A. Alnaser, Ragab Abouzeid, Ahmed Nabhan

**Affiliations:** 1 Mechanical Engineering Department, College of Engineering and Technology, Arab Academy for Science, Technology and Maritime Transport, Sadat Road, Aswan 81511, Egypt; 2 Mechanical Engineering Department, College of Engineering, King Saud University, Riyadh 11421, Saudi Arabia; 3 Center of Excellence for Research in Engineering Materials (CEREM), King Saud University, Riyadh 11421, Saudi Arabia; 4 School of Renewable Natural Resources, Louisiana State University AgCenter, Baton Rouge, LA 70803, USA; 5 Department of Production Engineering and Mechanical Design, Faculty of Engineering, Minia University, Minia 61519, Egypt

**Keywords:** rice straw nanofiber (RSNF), HDPE nanocomposites, biomedical applications, green composites

## Abstract

The efficient utilization of rice waste has the potential to significantly contribute to environmental sustainability by minimizing the waste impact on the environment. Through repurposing such waste, novel materials can be developed for various biomedical applications. This approach not only mitigates waste, but it also promotes the adoption of sustainable materials within the industry. In this research, rice-straw-derived nanofibers (RSNFs) were utilized as a reinforcement material for high-density polyethylene (HDPE). The rice-straw-derived nanofibers were incorporated at different concentrations (1, 2, 3, and 4 wt.%) into the HDPE. The composites were fabricated using twin-screw extrusion (to ensure homogenous distribution) and the injection-molding process (to crease the test samples). Then, the mechanical strengths and frictional performances of the bio-composites were assessed. Different characterization techniques were utilized to investigate the morphology of the RSNFs. Thermal analyses (TGA/DTG/DSC), the contact angle, and XRD were utilized to study the performances of the HDPE/RSNF composites. The study findings demonstrated that the addition of RSNFs as a reinforcement to the HDPE improved the hydrophilicity, strength, hardness, and wear resistance of the proposed bio-composites.

## 1. Introduction

As the global community seeks to create a more sustainable society that prioritizes environmental preservation and reduces reliance on fossil fuels, an increasing number of researchers are turning their attention to developing natural materials from waste products, such as rice straw (RS). This is reflected in the growing number of scientific studies that focus on this topic [1]. In line with this goal of utilizing natural resources more efficiently, the extraction of lignocellulose biomass from materials such as rice straw is an area of growing interest [2]. Rice straw is composed of naturalistic and environmentally friendly components, such as hemicellulose, lignin, and cellulose. In recent times, the utilization of bleached cellulose pulps devoid of lignin as the starting material for the isolation of rice straw nanofibers (RSNFs) has garnered considerable interest. This approach offers advantages, such as high yields, cost-effectiveness, and environmentally friendly characteristics [3]. The exploration of advanced bio-nanocomposite materials has also received significant attention, particularly in biomedical applications. HDPE and UHMWPE have been extensively employed as joint replacement materials for over 50 years, owing to their biocompatibility (use in different biomedical applications), self-lubrication, and excellent wear properties. As a result, they have become the primary bearing materials in artificial joints [4,5,6].

Tribology is a multidisciplinary field of study that focuses on understanding and analyzing the interactions between surfaces in relative motion. It encompasses various aspects, including friction, wear, and lubrication. By investigating these phenomena, tribology aims to enhance the understanding of surface interactions and develop strategies to minimize friction, reduce wear, and improve lubrication efficiency [7]. Despite it being a common occurrence in everyday life, most people are unfamiliar with the term. Bio-tribology is a specialized branch of tribology that focuses on the study of surface interactions within the organic components of the human body or living organisms. It encompasses the understanding of friction, wear, and lubrication in biological systems. In the context of artificial joints, the tribological performance is of paramount importance, as wear is a critical factor that impacts the longevity of the implant. Due to wear-related concerns, the lifespan of an artificial joint is typically limited to approximately 10–15 years. Therefore, bio-tribology plays a crucial role in developing materials, coatings, and lubrication techniques to minimize the wear and improve the performance and durability of artificial joints in biomedical applications [8]. In recent times, interest has increased among researchers in exploring the tribological properties of composite polymers reinforced with natural fibers, which is referred to as green tribology [9]. The investigation of the frictional behavior of naturalistic materials in reinforced composites is crucial for gaining a deeper understanding of their mechanical performances in various engineering components. By studying the frictional properties of such composites, researchers aim to evaluate their suitability, durability, and overall performance in practical applications [10,11,12,13].

Biopolymers have gained a lot of interest from many researchers for their potential use in various medical applications, such as dental implants, hip joint replacement, and bone cement [14,15,16,17,18,19]. The addition of nanofillers is an important approach that is utilized to enhance the different characteristics of polymers. Nanofillers, such as particles or fibers, have shown compatibility with different polymer matrices, which can enhance their chemical, mechanical, and physical properties, making them a suitable partner for various medical applications. In addition, some research has been conducted on the tribological properties of composites made from natural fibers, such as kenaf [20,21], oil palm [21,22], bamboo [23], curaua fiber [24], and jute [25], combined with polymers. However, the tribological behavior of HDPE/RSNF bio-nanocomposites still needs more study.

The focus of this paper is on the development of next-generation bio-nanocomposites utilizing natural materials. These composites are aimed at achieving an improved tribological performance, including lower friction coefficients, reduced wear, and enhanced mechanical properties. In order to achieve the desired objectives, HDPE/RSNF nanocomposites were fabricated after utilizing a twin-screw-extruder machine. This manufacturing technique was chosen to ensure a homogenous distribution of RSNFs within the HDPE matrix. Varying proportions of RSNFs were incorporated into the composites during the extrusion process. Subsequently, injection molding was employed to shape the dry blends into the desired final form. To evaluate the produced nanofiller and nanocomposites, various techniques were employed to assess the morphology of the produced nanofibers in addition to the HDPE/RSNF bio-composite characteristics. Additionally, the nanocomposite mechanical and tribological characteristics were examined.

## 2. Experimental Work

### 2.1. Nanocomposite Preparation

In this study, high-density polyethylene (HDPE) was sourced from Sigma-Aldrich (Saint-Quentin-Fallavier, France). The HDPE came as a fine powder with a particle size ranging from 40 to 90 µm and a density around 0.94 g/cm^3^. Other supportive materials, such as sodium stearate (CH3(CH2)16COONa ≥ 92, Roth France, LAGNY SUR MARNE, France) and MAPE, with a viscous property of 500 cP at 140 °C (as per the literature), were utilized. These materials were selected for their specific properties and suitability for the fabrication of the HDPE/RSNF nanocomposites. The raw material for rice straw used in the study was sourced from a native farm in Egypt. In order to prepare the straw for further processing, it was cleaned by washing it with tap water and subsequently dried in ambient air. Reagent-grade chemicals, including sodium carbonate, sulfite, and chlorite (with a concentration of 80%), in addition to glacial acetic acid, were obtained from Fisher Scientific (Loughborough, UK).

To obtain RS nanofibrils, unbleached naturalistic sulfite pulp was made via pulping RS employing a 10% sodium sulfite solution at a constant temperature of 160 °C for 2 h. This pulping process has been previously described in reference [3]. Following the pulping process, chemical analyses were conducted on the prepared pulps to determine their various contents. These analyses provide valuable information regarding the chemical structures and characteristics of rice straw pulps, aiding in the understanding of their suitability for further processing and potential applications [26]. Subsequently, the prepared pulp was subjected to a bleaching process for a duration of 1 h. This bleaching process involved the use of a mixture comprising sodium chlorite and acetic acid, and it was conducted at a temperature of 80 °C [4]. After the bleaching process, the pulp suspension was further refined using a disk refiner, specifically a Valley Beater. The refinement process involved passing the pulp through the refiner at a consistency of 2% weight. The refining was carried out until a level of 90° SR, according to ISO 5267-1, was achieved. To ensure accuracy and reliability, measurements were taken at least three times during the refining process. This refining step helped to improve the properties and quality of the pulp, leading to the production of refined and well-processed rice straw nanofibrils. The extraction of RSNFs based on the bleached pulp was performed using a Super mass collider ultra-fine friction grinder (MKZA6-2, Masuko Sangyo Co., Ltd., Kawaguchi, Japan) with a recirculation mechanism attached. The extraction approach followed a previously established method. In this protocol, the grinder is operated in a loop mode, with each loop equivalent to 10 passes. The fibrillation process was conducted using discs with ranges of 0, −5, and −10 μm with a speed of 1500 rpm. The combination of the disk gaps and rotation speed facilitated the breakdown of the rice straw fibers into nanoscale dimensions, resulting in the production of rice straw nanofibers suitable for further characterization and application in various fields. The RSNF suspension was poured inside a cylindrical die and was then left in contact with the lyophilized shelves at a temperature of −50 °C under ambient pressure for 2 h. Following the fibrillation process, the temperature was gradually raised from −50 up to 20 °C over 24 h. This temperature increase was carried out under a vacuum condition of 0.1 mbar. Once the desired temperature of 20 °C was reached, it was maintained at a constant level for a duration of 30 min while reducing the applied pressure to 0.01 mbar.

The synthesis of the composite samples involved three mechanical steps: two steps were conducted utilizing a twin-screw-extruder machine, while the final step was conducted using an injection-molding machine. These steps have been previously established in the literature as an effective method for creating polymer composites [27,28]. The synthesis process utilized two phases of the extrusion process. In the first phase, the masterbatch was compounded, which involved the incorporation of the desired additives and reinforcement materials into the base polymer (HDPE in the current study). This step ensured a homogenous distribution of the RSNFs within the HDPE resin. In the second step, the extruded nanocomposite masterbatch was diluted. This step involved the further processing of the masterbatch to obtain the required composition and properties of the final composite material. The extrusion process is essential, as it facilitates the dispersion of the RSNFs within the polymer matrix, resulting in a uniform and well-mixed composite material. The production of nanocomposites involved incorporating the freeze-dried RSNFs as the nanofiller, along with a constant content of MAPE as a synchronizer and a constant amount of sodium works as a plasticizer. The extruder was equipped with six heating zones, which operated with a controlled temperature ranging from 200 to 230 °C. This temperature gradient was carefully controlled to ensure the proper melting and blending of the components, resulting in a homogenous mixture of HDPE, RSNFs, MAPE, and sodium stearate. To ensure consistency and comparability, multiple nanocomposites with varying contents were prepared using the same extrusion process. The RSNFs along with the HDPE were inserted inside the extruder, where they were subjected to a recirculation looping extrusion for 10 min. The extrusion process involved maintaining a constant screw speed of 200 rpm and a manufacturing temperature ranging from 200 to 230 °C. To ensure a fair comparison, the HDPE samples underwent the same extrusion procedure as the nanocomposites, with equal amounts of MAPE and sodium stearate added to all samples.

The HDPE/RSNF nanocomposites were created with 0 wt.% (HDPE-00), 1 wt.% (HDPE-01), 2 wt.% (HDPE-02), 3 wt.% (HDPE-03), and 4 wt.% (HDPE-04) of RSNFs. These nanocomposites were produced by diluting the masterbatch with HDPE and an additional material: 5 wt.% MAPE. Sodium stearate was used as a lubricant at 4 wt.% weight. Then, the extrusion process, using a twin-screw DSM-Xplore15cc Micro-extruder (Xplore instruments BV, Sittard, The Netherlands) and an injection-molding machine Haake MiniLab II, (Thermo Fisher Scientific, Karlsruhe, Germany), was conducted again to produce HDPE nanocomposites with different weight fractions of RSNFs. After the extrusion process, the resulting materials were further processed (pelletizing) using a HAAKE Minijet II system. The injection-molding machine was set to a temperature of 200 °C to melt the material (details of injection molding conditions are listed in Table 1). Once melted, the material was injected into a mold under a pressure of 550 bar for a duration of 15 s. After the injection-molding process, the samples were taken to be tested. The sample like “dog bone” specimens for the tensile tests and discs with dimensions of 25 mm × 1.5 mm were obtained.

### 2.2. Characterization and Testing

X-ray diffraction data were recorded for the nanocomposite samples at room temperature using a P-analytical diffractometer. The X-ray diffraction analysis was conducted using an instrument operating at a voltage of 45 kV and a current of 30 mA. The instrument was equipped with a copper K-alpha anode, which emitted X-rays for the diffraction measurements. The crystallinity index (CI) was determined employing Equation (1), which involved analyzing the zones of the crystalline peaks in addition to the amorphous halos in the obtained XRD patterns [29]:

(1) CA%=A CA total×100

To investigate the wettability of the HDPE/RSNF nanocomposites, the contact angle was estimated utilizing OCA 40 Micro. The measurement used a 2 μL droplet of solvent in DI water, and contact angles were detected after imaging. The measurements were taken at a constant temperature of 22 °C, and the humidity was around 45%. The video used in the measurement process was recorded at 72 frames/sec to allow for the precise observation of any slight adjustments to the droplet on the surface. This method is able to compute contact angles with a precision of 0.1 degrees.

The mechanical performances of the produced composites were evaluated based on identifying the tensile strength, break elongation, and modulus of elasticity applying the ASTM D638 standard. The measurements were performed utilizing an Instron 5965 tension machine, which has a load cell with a 5 kN capacity. The initial displacement between the pneumatic jaws was adjusted at 10 mm, and the tension rate was adjusted at 2 mm/s. The test was performed on five specimens.

The hardness of the injection HDPE nanocomposite specimens was estimated according to the ASTM D2240 standard. During the test, a standardized force was applied using a uniform indentation head, resulting in an indentation on the HDPE composite surface. To determine the hardness of the nanocomposite samples, the indentation depths were identified on five specimens, and in five different locations on each specimen, and the mean hardness was calculated.

The thermal characteristics of the HDPE nanocomposites were analyzed utilizing a PerkinElmer DSC (Differential Scanning Calorimetry) instrument. The specimens were subordinated to heating–cooling–heating periods in the presence of nitrogen. The rate of heat change was adjusted at 10 °C/min, and the variation in temperature during the cycle ranged from −100 to 250 °C. The DSC instrument measured the heat flow and temperature changes of the specimens, providing information about their thermal properties and behavior.

Nanocomposite specimens were tested according to ASTM G99 in a dry circumstance to determine their friction coefficients and wear characteristics. The tribometer utilized a stainless-steel counter-face disc with a diameter of 180 mm. The roughness of the counter-face disc was determined, and it was 0.023 μm. During the experiment, the HDPE nanocomposites were represented as pins in a cylindrical shape. The frictional characteristics for each HDPE nanocomposite specimen were evaluated with variation in the standard normal force (2, 4, 6, 8, and 10 N) at a constant speed. The friction coefficient averages were reported. Wear rate was measured (mass loss) by recording nanocomposite samples before and after the frictional test utilizing an electronic balance (0.0001 g). After conducting the tribological tests, the surfaces of the nanocomposite specimens were inspected utilizing optical microscopy (OLYMPUS BX53M, De Witt, IA, USA) and scanning electron microscopy (Jeol’s benchtop SEM JSM-6000).

## 3. Results and Discussion

**Figure 1:**
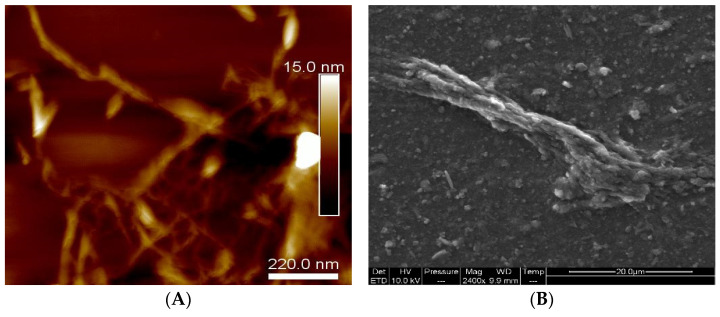
The morphology of rice straw nanofibers using (**A**) AFM and (**B**) SEM.

**Figure 2:**
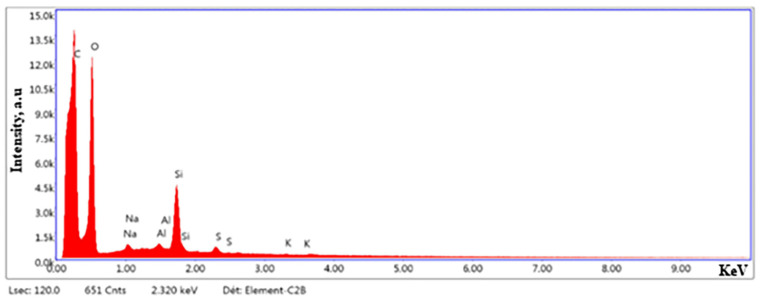
EDS analysis of rice straw nanofibers (RSNFs).

The morphology of the RSNFs extracted from rice straw was analyzed using scanning electron microscopy and atomic force microscopy. The AFM (Figure 1A) revealed the presence of uniform, nanosized fibers extracted from the RS pulp utilized in the ultra-fine grinder. The images were obtained from highly diluted air-dried suspensions (0.001, *w*/*w*%) deposited on freshly glued mica surfaces. The SEM displayed in Figure 1B clearly exhibits the presence of silica in the samples, which was further confirmed by EDS analysis (Figure 2). The identification of residual silica in the rice straw pulp can be attributed to the existence of nanosized spherical parts called organic silica inside the bleached rice straw [30].

**Figure 3:**
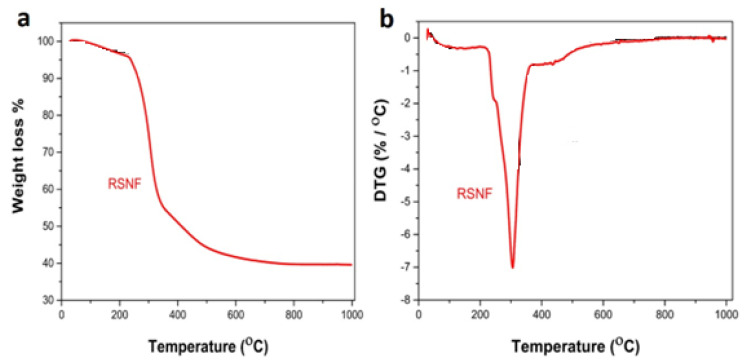
Thermal analysis of rice straw nanofibers (RSNFs): (**a**) TGA and (**b**) DTG.

The thermal stability of RSNFs is an essential parameter that greatly influences the quality of the nanocomposites produced using a melt-mixing technique, such as extrusion and injection molding. TGA and DTG were employed to study the RSNF thermal stability, as depicted in Figure 3a,b. The first degradation stage began at 230 °C. The second degradation phase took place at temperatures of 340 °C [31]. Lastly, when the temperatures exceeded 500 °C, the aromatic rings of the RSNFs began to dissolve. The concentration of the RSNF residuals was found to be 40%. The three previous thermal degradation stages of cellulosic nanofibers are considered the standard melting procedure for HDPE-based materials [32,33].

**Figure 4:**
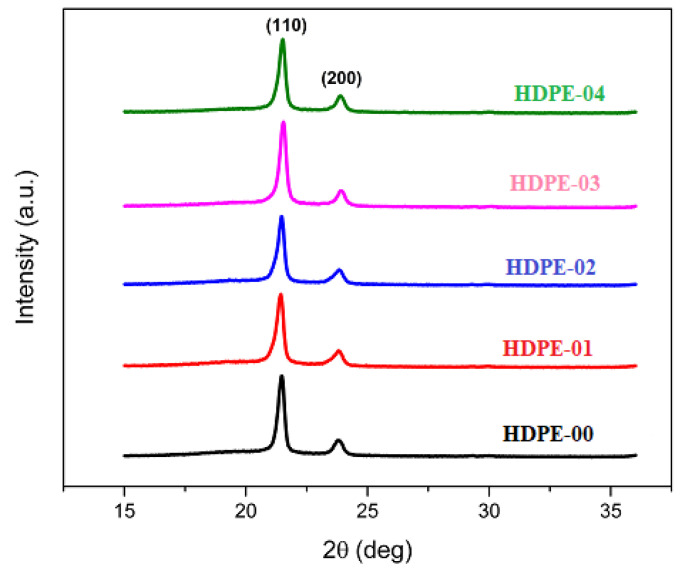
XRD of HDPE/RSNF nanocomposites.

X-ray diffractograms were utilized to assess the crystallinity of the fabricated HDPE/RSNF nanocomposites. The obtained X-ray diffractograms are depicted in Figure 4, providing insights into the crystalline properties of the composites. The X-ray diffractograms exhibit two prominent peaks corresponding to the diffraction planes (110) and (200) of the orthorhombic unit cell. These peaks were observed at approximately 21.46° and 23.8°, respectively [34]. The presence of these peaks in the RSNFs provides evidence of a semi-crystalline manner, indicating the coexistence of both amorphous and crystalline zones in the cellulose [35]. By increasing the RSNF filler content in the HDPE nanocomposites, subtle changes in the diffraction patterns of the (110) and (200) planes were observed. However, after reaching a certain threshold of nanocellulosic fiber content, the diffraction manner did not change across any of the nanocomposite specimens. The observed inconsiderable change in the diffraction peaks with the increasing RSNF content suggests that the addition of RSNF fibers did not significantly alter the structure of the HDPE. The orthorhombic nature of the HDPE remained unchanged, indicating that the presence of RSNFs did not have a considerable effect on the overall crystal structure of the nanocomposites. Notably, there was an overlapped peak observed at approximately 22° in 2θ, representing the presence of cellulose nanofibers in conjunction with HDPE. This observation aligns with previous studies involving nanocellulose fillers within a polyethylene matrix [36,37].

**Figure 5:**
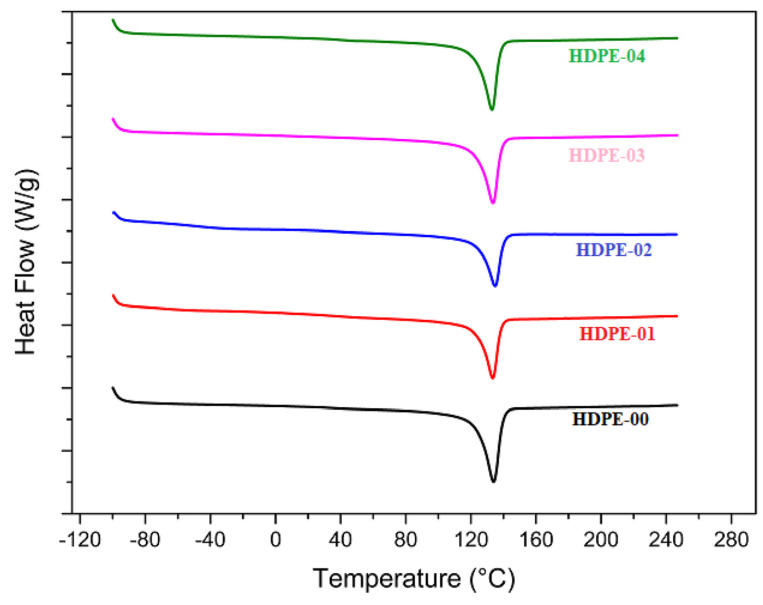
DSC of HDPE/RSNF nanocomposites.

The thermal properties of the extruded nanocomposites are displayed in Figure 5 as DSC thermograms. It is obvious that there were minimal variations in the melting temperature and solidification temperature of the HDPE/RSNF nanocomposites compared with pure HDPE, indicating that the HDPE/RSNF nanocomposite thermal stability did not change. Nevertheless, and as previously observed in the XRD analysis, the crystallinity of the HDPE/RSNF nanocomposites increased, although DSC is not as accurate as XRD in determining the degree of crystallinity in polymers.

**Figure 6:**
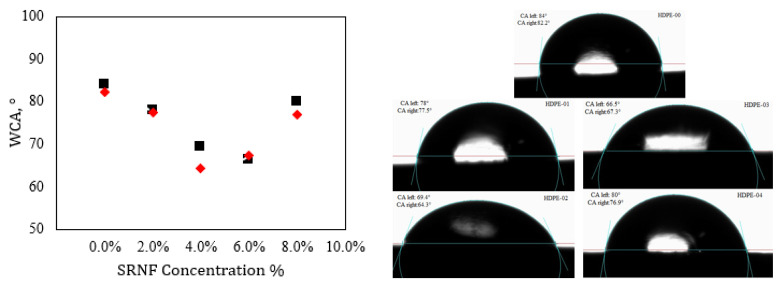
WAC of HDPE/RSNF nanocomposites.

The average results of the WCA measurements for the pristine HDPE and RSNF composites with 0, 1, 2, 3, and 4 wt.% nanofiller contents are represented in Figure 6. It is obvious that in comparison to the pristine sample, all percentages of nanofiller had an effect on the wetting properties of the samples. The WCA values for the RSNF nanocomposite samples varied from 64° to 80°, indicating that the inclusion of cellulosic nanofibers greatly enhanced the hydrophilicity. This occurred due to the exitance of cellulose, which has a hydrophilic nature, in addition to the presence of hydroxyl groups in cellulose [38]. Therefore, all nanocomposite samples are more hydrophilic than HDPE. Additionally, the improved hydrophilicity of the HDPE/RSNF samples facilitated cell adhesion and growth [39,40].

**Figure 7:**
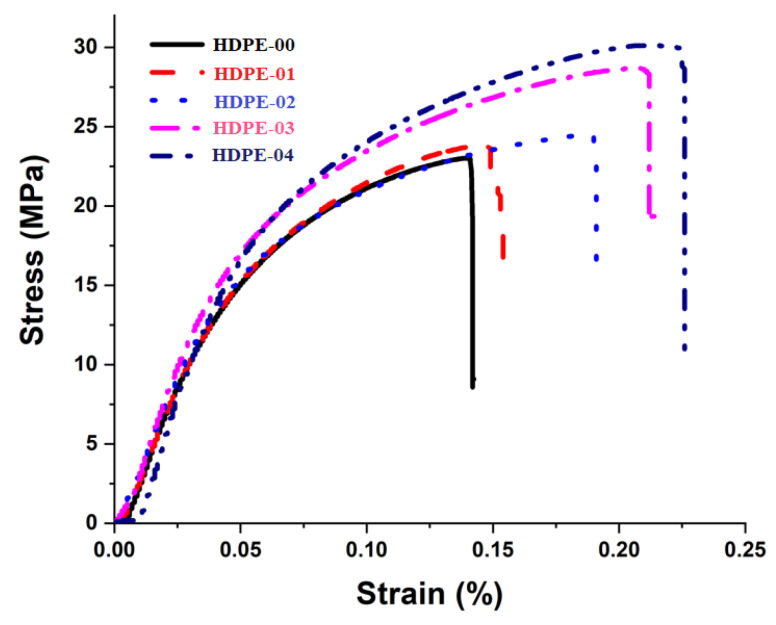
Stress–strain curves of HDPE/RSNF nanocomposites.

**Figure 8:**
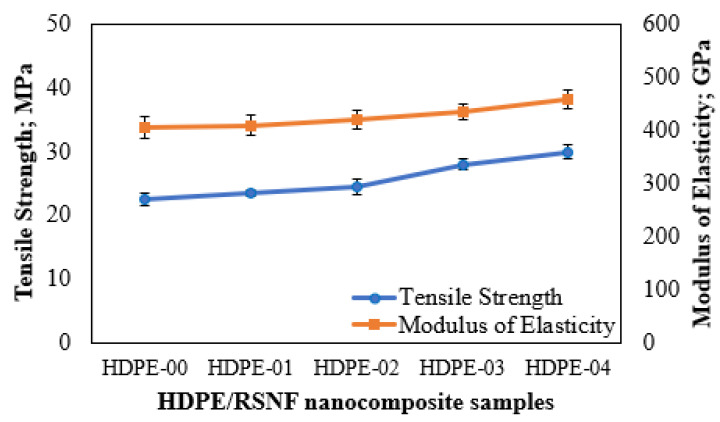
Changes in the mechanical performances of the HDPE/RSNF nanocomposites.

The mechanical performances of the fabricated HDPE/RSNF nanocomposites were compared to the performance of pure HDPE, as seen in the stress–strain curves in Figure 7, and the extracted data are shown in Figure 8. The tensile test outcomes revealed that the nanocomposites containing RSNFs had higher average tensile strengths. The study found that adding RSNFs to the HDPE resulted in a continuous improvement in the properties from 1 to 4 wt.% compared to pure HDPE, and the tensile strengths of the HDPE-01, HDPE-02, HDPE-03, and HDPE-04 nanocomposites increased by 7.4%, 15.3%, 21.6%, and 30%, respectively. In addition to the improvements in the tensile strengths, the HDPE/RSNF nanocomposites also exhibited significant enhancement in elasticity and elongation when compared to the HDPE-00 specimen. The improved mechanical performances of the HDPE/RSNF nanocomposites can be attributed to two key factors. Firstly, the employment of a coupling agent, MAPE, helped in enhancing the interfacial adhesion between the HDPE and RSNF filler [41]. This improved interfacial adhesion leads to better stress transfer and load distribution, resulting in enhanced mechanical properties. Secondly, the efficient distribution of RSNFs in the HDPE through the twin-screw-extrusion process improved the mechanical performance. The extrusion process ensures a uniform distribution of RSNFs throughout the HDPE, reducing the presence of agglomerates or voids. This uniform dispersion enhances the overall strength, stiffness, and toughness of the nanocomposite material. [42,43].

**Figure 9:**
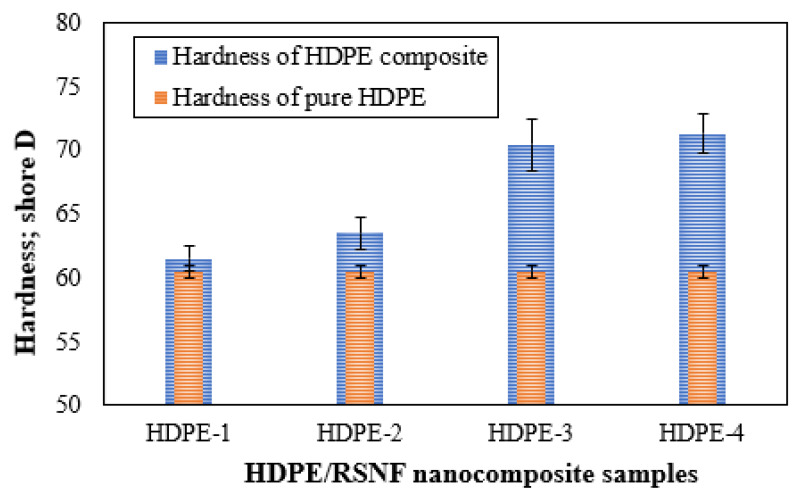
Hardness of HDPE/RSNF nanocomposites.

Hardness, which represents a material’s ability to withstand pressure, is a significant mechanical attribute to consider when evaluating composites, especially when they are used in applications such as artificial joints [44]. Figure 9 shows the variation that occurred in the Shore D hardness of pure HDPE and HDPE/RSNF nanocomposites. It is clear that the hardness of the HDPE increased as the weight fraction of RSNFs was added up to 1.5%. However, when the weight fraction was increased to 2%, there was only a slight increase in hardness observed.

**Figure 10:**
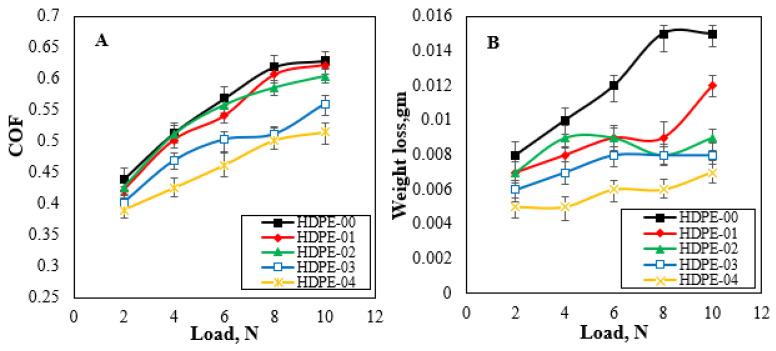
(**A**) Changes in the friction coefficient and (**B**) wear of HDPE/RSNF nanocomposites.

To investigate the tribological behavior of the proposed HDPE/RSNF nanocomposites, frictional tests were carried out. The tribological tests were conducted under different loads (2, 4, 6, 8, and 10 N) at a constant frictional speed. The results depicted in Figure 10A show the variation in the friction coefficient under different loads for all the HDPE/RDNF composites. The ability to withstand wear is a crucial factor in determining the lifespan of friction materials [45]. As shown in Figure 10B, the wear-loss values for the HDPE/RSNF nanocomposites were assessed to quantify their wear resistances.

It is typical for the friction coefficient and wear loss to rise as the normal load increases. However, the utilization of RSNFs resulted in considerable reductions in both the wear loss and friction coefficients of the HDPE/RSNF nanocomposites. Table 2 shows the influence of adding RSNFs to the HDPE: there is a notable improvement in the tribological characteristics. The enhancement in the mechanical and tribological characteristics of HDPE by augmenting its strength and enabling the RSNFs to handle stress has been highlighted in various studies [46]. Additionally, the high surface area–volume ratio of RSNFs plays a crucial role in enhancing the particle bonding within HDPE/RSNF composites. This improved bonding leads to increased interfacial adhesion between the RSNFs and the HDPE matrix, thereby improving the overall mechanical and tribological properties of the nanocomposite. The enhanced particle bonding contributes to improved characteristics, such as hardness and crystallinity, which are key factors in determining the composite resistance to wear and friction. The increased interfacial adhesion also helps in distributing the applied load more evenly, reducing localized stress concentrations and preventing premature failure or damage [47]. Furthermore, the high hydrophilicity of RSNFs also helps reduce the coefficient of friction [48]. As seen in Table 2, the addition of RSNFs to the HDPE matrix also improved the hydrophilicity of the samples. Rice straw fibers contain a high amount of silica [49], which may also contribute to the improved tribological characterization [50] of nanocomposite materials.

**Figure 11:**
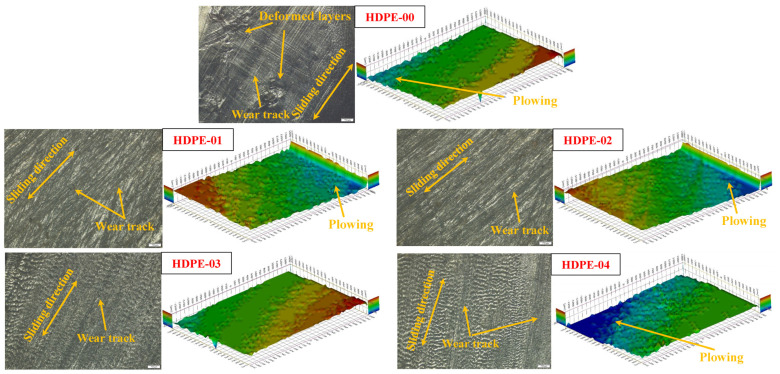
Microscopic inspection of the HDPE/RSNF nanocomposite surfaces.

Upon examining the surfaces of the HDPE nanocomposites after friction, as depicted in Figure 11, we observed that they exhibited a finer texture compared to the surface of the pure HDPE (HDPE-00). This observation suggests that the nanocomposites experienced lower wear loss in comparison to HDPE-00. The 3D outcome images of the ribbed surfaces (Figure 11) further support the measured values. Overall, the incorporation of RSNFs into the HDPE matrix not only improved the mechanical properties but also enhanced the wear resistance of the HDPE/RSNF nanocomposites when compared to the surfaces of the neat HDPE without the nanofibers. This highlights the potential of RSNFs as an effective reinforcing agent for improving the tribological properties of HDPE.

**Figure 12:**
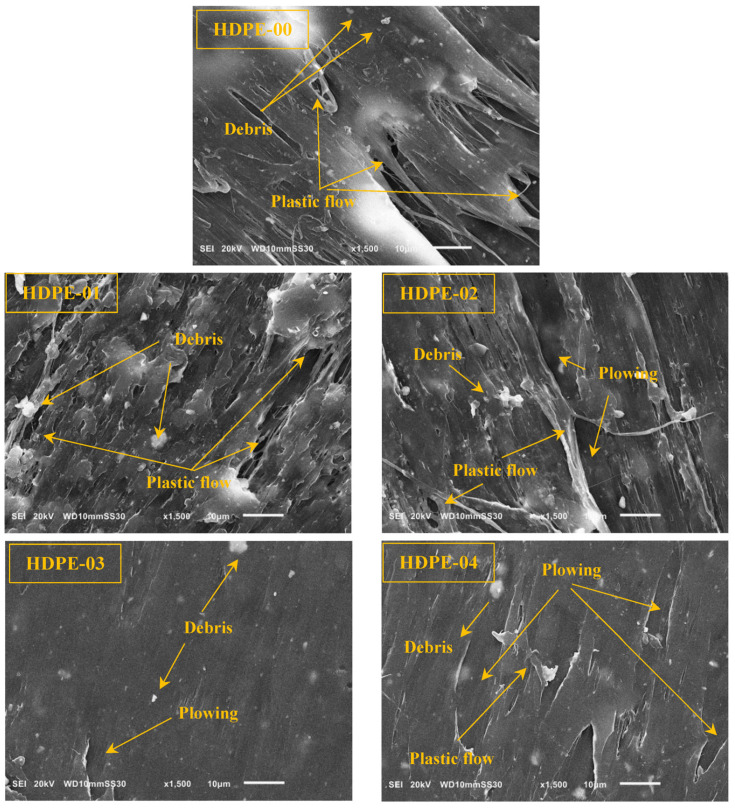
SEM images of the HDPE/RSNF nanocomposite worn surfaces.

To further evaluate the worn surfaces in more detail, SEM images were taken, as shown in Figure 12. The worn surface of the pure sample (HDPE-00) appears significantly damaged, with areas of plastic deformation and plowing. In contrast, the SEM images reveal that the surfaces of the nanocomposite samples exhibit less damage than the pure HDPE sample. This implies that the RSNF filler contributed to the improvement in the strength and resistance of the HDPE nanocomposites. The worn surface of the sample HDPE-03 presents a good and smooth surface, suggesting that this composition of RSNF filler is the optimal choice to use with HDPE.

## 4. Conclusions

The current research presents a comprehensive methodology for the production of rice straw nanofibers. The produced nanofibers were thoroughly characterized to ensure their suitability for use as a reinforcement for high-density polyethylene in biomedical applications. The fabrication process for HDPE/RSNF nanocomposites was also carried out to guarantee homogenous filler distribution within the HDPE matrix. The mechanical and tribological characteristics of the HDPE/RSNF nanocomposites were assessed, and the results presented a noticeable improvement in the nanocomposite strength, stiffness, and wear. The study revealed that adding RSNFs to HDPE matrices contributes to enhancing various properties: the tensile strength, COF, and wear rate of the HDPE/RSNFs filled with 4% wt. increased about 30%, 17.5%, and 50%, respectively, compared to pure HDPE. An analysis of the worn surfaces after the friction process revealed a strong correlation between the tribological performance of the HDPE and the presence of RSNFs. This research highlights the potential of RSNFs for use as a unique filler for HDPE in biomaterial applications, and it provides a valuable reference for further research in this field.

## Figures and Tables

**Table 1:** Injection molding conditions.

Injection-Molding Conditions	
Injection temperature	200 °C
Mold temperature	65 °C
Injection speed (velocity)	100 rpm
Injection time	15 s
Hold time	9 s
Hold pressure	550 bar

**Table 2:** Tribological performance enhancement of HDPE/RSNF nanocomposites compared to pure HDPE.

Load (N)	Friction Coefficient (%)				Wear (%)			
	HDPE-01	HDPE-02	HDPE-03	HDPE-04	HDPE-01	HDPE-02	HDPE-03	HDPE-04
2	3.8	3	8.5	11.3	12.5	12.5	25	37.5
4	2.2	0.5	8.7	17.3	20	10	30	50
6	5	2	11.5	19	25	25	33.3	50
8	2.013	5.4	17.4	19	40	46.7	46.7	60
10	1.1	4	11	18.2	20	40	46.7	53.3

## Data Availability

Not applicable.
